# Pathologies auriculaires et périauriculaires au service d’ORL du Centre hospitalier universitaire Sylvanus Olympio de Lomé (Togo)

**DOI:** 10.48327/mtsi.v4i2.2024.524

**Published:** 2024-06-20

**Authors:** Essobizou AMANA, Gbandi PEKOULA, Winga FOMA, Djim Hervey REOULEMBAYE, Comí Rémy ZEYI, Bathokédéou AMANA

**Affiliations:** Service d’oto-rhino-laryngologie (ORL) et de chirurgie cervicofaciale, Centre hospitalier universitaire Sylvanus Olympio, 198, Rue de l’Hôpital Tokoin Hôpital, BP 57 Lomé, Togo

**Keywords:** Auricule, Lobule de l’oreille, Chéloïde, Othématome, Hôpital, Togo, Afrique subsaharienne, Auricle, Ear lobule, Keloid, Othematoma, Hospital, Togo, Sub-Saharan Africa

## Abstract

**Objectif:**

L’objectif de cette étude est de décrire les aspects épidémiologiques, les caractéristiques cliniques et les principes de prise en charge des différents types de pathologies auriculaires et périauriculaires.

**Patients et méthode:**

Il s’agit d’une étude rétrospective descriptive de 5 ans (1^er^ mai 2018 au 30 avril 2023) portant sur les dossiers des patients ayant consulté pour une plainte fonctionnelle ou esthétique se rapportant à l’auricule ou à la région périauriculaire dans le service d’ORL et de chirurgie cervico-faciale du Centre hospitalier universitaire Sylvanus Olympio.

**Résultats:**

Un effectif de 159 cas sur 5 ans, soit une fréquence annuelle de 31 cas, a répondu aux critères d’étude. L’âge moyen des patients était de 22,2 ans. Les enfants et les étudiants représentaient respectivement 24,5 % et 23,9 % des cas. Les pathologies auriculaires représentaient 64,8 % des cas et les périauriculaires 36,2 % des cas. Les tumeurs et les traumatismes représentaient respectivement 33,3 % et 29,6 % et les pathologies congénitales étaient retrouvées dans 29,9 % des cas. Parmi les lésions traumatiques, les coups et blessures volontaires étaient en cause dans 21,3 % des cas, suivies des accidents de la voie publique dans 17,2 %. L’oreille droite était atteinte dans 48 % et le lobule dans 40,4 %. Les chéloïdes représentaient 17,6 % de l’ensemble et 53 % des tumeurs et pseudotumeurs; le siège gauche était retrouvé dans 50 % des cas. Le piercing était la cause des chéloïdes dans 10,7 %.

**Conclusion:**

Les pathologies auriculaires et périauriculaires étaient dominées par les tumeurs bénignes, les traumatismes et les pathologies congénitales et intéressaient les sujets jeunes. La prise en charge est faite selon le type de lésion, dans un souci fonctionnel et esthétique.

## Abstract (en)

**Objective:**

The aim of this study is to describe the epidemiological aspects, clinical features and principles of management of different types of auricular and peri-auricular pathologies.

**Patients and Methods:**

This is a 5-year retrospective descriptive study (from May 1, 2018 to April 30, 2023) of the records of patients who consulted for a functional or cosmetic complaint relating to the auricle or periauricular region in the ENT and cervico-facial surgery department of the Centre hospitalier universitaire Sylvanus Olympio.

**Results:**

A total of 159 cases over 5 years, i.e. an annual frequency of 31 cases, met the study criteria. The mean age of the patients was 22.2 years. Children and students accounted for 24.5% and 23.9% of cases respectively. Auricular pathologies accounted for 64.8% of cases and peri-auricular pathologies for 36.2%.

Tumors and trauma accounted for 33.3% and 29.6% of cases respectively, and congenital pathologies were found in 29.9% of cases. Among traumatic lesions, intentional assault and battery was the cause in 21.3%, followed by road accidents in 17.2%. The right ear was affected in 48% and the lobule in 40.4%. Keloids accounted for 17.6% of all cases, and 53% of tumors and pseudotumors. The left ear was involved in 50% of cases. Piercing was the cause of keloids in 10.7% of cases.

**Conclusion:**

Auricular and peri-auricular pathologies were dominated by benign tumors, trauma and congenital pathologies, and involved young subjects. Management is based on the type of lesion, with functional and aesthetic considerations in mind.

## Introduction

Les pathologies auriculaires et périauriculaires sont l’ensemble des atteintes du pavillon de l’oreille et de ses environs, quelle que soit son origine [9]. Ces atteintes peuvent être traumatiques, infectieuses, inflammatoires, tumorales ou encore congénitales [2, 8]. Le diagnostic est généralement clinique, mais il est nécessaire de rechercher les lésions associées dans la sphère ORL ou à distance, surtout dans les contextes traumatiques et malformatifs. Ces pathologies mettent en jeux le pronostic fonctionnel et entraînent également un préjudice esthétique [10]. Au Togo, aucune étude intéressant spécifiquement le pavillon de l’oreille et ses environs n’a encore été réalisée, et la littérature scientifique sur la question est également pauvre. Ainsi, l’objectif de ce travail est de passer en revue les aspects épidémiologiques et les caractéristiques cliniques des différents types de pathologies auriculaires et périauriculaires au service d’otorhinolaryngologie et de chirurgie cervico-faciale du Centre hospitalier universitaire Sylvanus Olympio (CHU SO) de Lomé.

## Patients et méthode

Il s’agit d’une étude rétrospective descriptive portant sur des pathologies auriculaires et périauriculaires au service d’ORL et de chirurgie cervico-faciale du CHU SO de Lomé. La période d’étude va du 1^er^ mai 2018 au 30 avril 2023, soit cinq ans. Les dossiers des patients ayant consulté pour une plainte fonctionnelle ou esthétique se rapportant à l’auricule ou à la région périauriculaire de l’oreille ont été inclus dans notre étude. N’ont pas été inclus les pathologies du conduit auditif externe, les atteintes périauriculaires au-delà du plan souscutané, comme les atteintes parotidiennes, ostéo-articulaires (l’articulation temporomandibulaire et os temporal) ou musculaires (le sterno cléido mastoïdien) et les dossiers incomplets. Les données sociodémographiques et cliniques ont été étudiées. Les données ont été traitées par le logiciel Epi info version 7.2.5.0. Un accord verbal des patients a été obtenu pour l’utilisation des photos.

## Résultats

**Figure 1:**
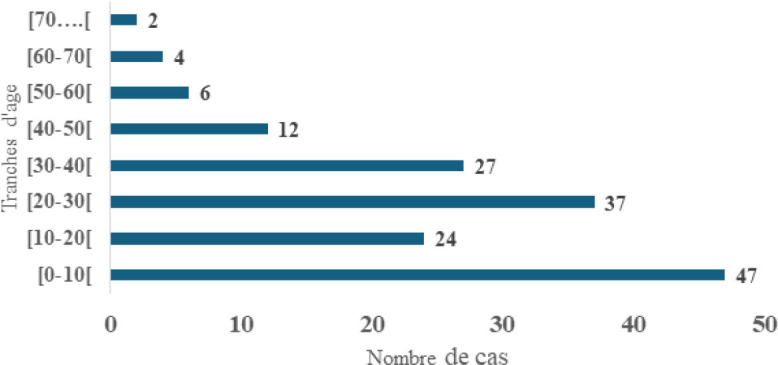
Répartition des patients selon les tranches d’âge Patient distribution by age group

Un effectif de 159 cas a été colligé, soit une fréquence de 31,8 cas par an. L’âge moyen des patients était de 22,2 ans, avec des extrêmes d’un mois et 80 ans. La tranche d’âge de zéro à 10 ans représentait 29,6 % des cas (Fig. 1). Le sexe féminin était concerné dans 58,5 % des cas, avec un sex-ratio de 0,70. Les pathologies étaient des tumeurs et pseudotumeurs dans 53 cas (33,3 %) et traumatiques dans 47 cas (29,6 %) (Tableau I). Selon le siège, elles étaient auriculaires dans 103 cas (64,8 %) et périauriculaire dans 56 cas (35,2 %)

**Tableau I:** Réparation des différentes pathologies auriculaires et périauriculaires Distribution of different ear and periauricular pathologies

	Effectif	%
**Tumorale et pseudotumorale**	53	33,3
**Traumatique**	47	29,6
**Congénitale**	38	23,9
**Infectieuse**	19	11,9
**Inflammatoire**	2	1,3
**Total**	**159**	**100**

Les lésions auriculaires intéressaient l’hélix dans 68 cas (66 %) et le lobule dans 27 cas (26,2 %). L’oreille droite était atteinte dans 49 cas (47,6 %) et il y avait une lésion cartilagineuse dans 20 cas (19,4 %) (Tableau II). Quant aux lésions périauriculaires, elles étaient préauriculaires dans 34 cas et rétroauriculaires dans 22 cas

**Tableau II:** Répartition des lésions selon leur siège auriculaire Distribution of injuries by auricular location

	Effectif	%
**Hélix**	68	66
**Lobule**	26	25,2
**Anthélix**	1	1
**Tragus**	1	1
**Tout le pavillon**	7	6,8
**Total**	**103**	**100**

**Figure 2:**
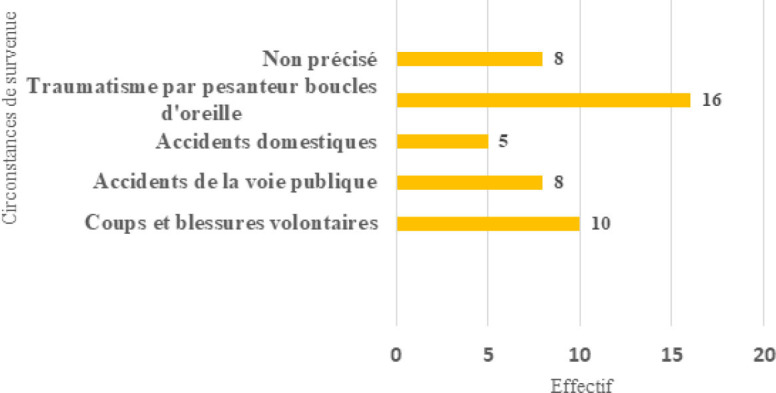
Répartition selon les circonstances de survenue du traumatisme Distribution according to the circumstances in which the trauma occurred

Les pathologies traumatiques représentaient 47 cas, soit 29,6 % des pathologies auriculaires et périauriculaires. Comme mécanisme de survenue, les coups et blessures volontaires (CBV) représentaient 10 cas (21 %), suivis des accidents de la voie publique (AVP) avec 8 cas (17 %) (Fig. 2).

Les lésions traumatiques étaient des plaies franches dans 25 cas (53 %), des déchirures du lobule par le poids des boucles d’oreille dans 16 cas (34 %) et des othématomes dans 6 cas (13 %).

**Figure 3:**
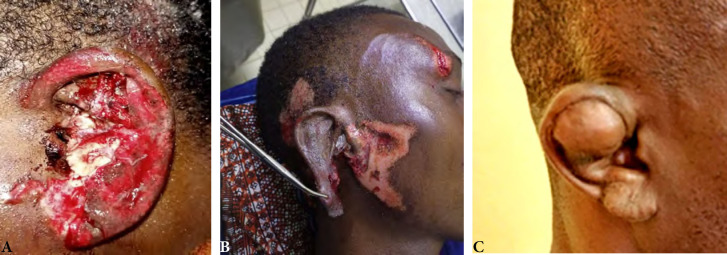
Lésions traumatiques auriculaires Traumatic ear injuries

Les lésions intéressaient le lobule dans 19 cas (41 %) (Tableau III) et l’oreille droite était atteinte dans 48 % des cas (Fig. 3 et 4). Au plan thérapeutique, le traitement a consisté en un parage et une suture des plaies ainsi que dans un drainage avec mise en place de bourdonnet dans six cas.

**Tableau III:** Répartition des lésions traumatiques selon leur siège auriculaire Distribution of traumatic injuries by auricular location

	Effectif	%
**Lobule**	19	41
**Hélix**	18	38
Tout le pavillon*	8	17
**Anthélix**	1	2
**Tragus**	1	2
**Total**	**47**	**100**

* Dans cinq cas la lésion s’étendait à la région péri-auriculaire

**Figure 4:**
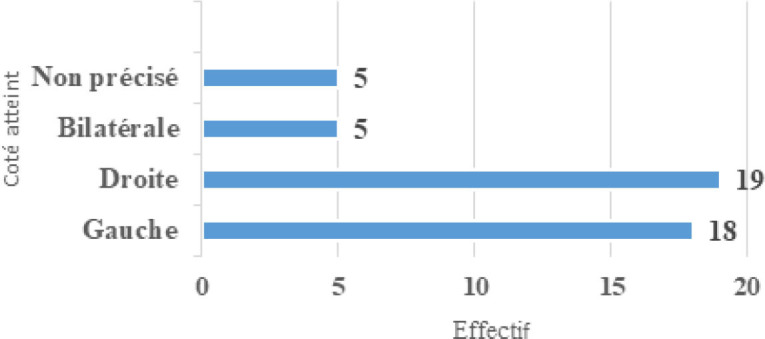
Répartition des lésions traumatiques en fonction du coté atteint Distribution of traumatic injuries according to the side affected

**Figure 5:**
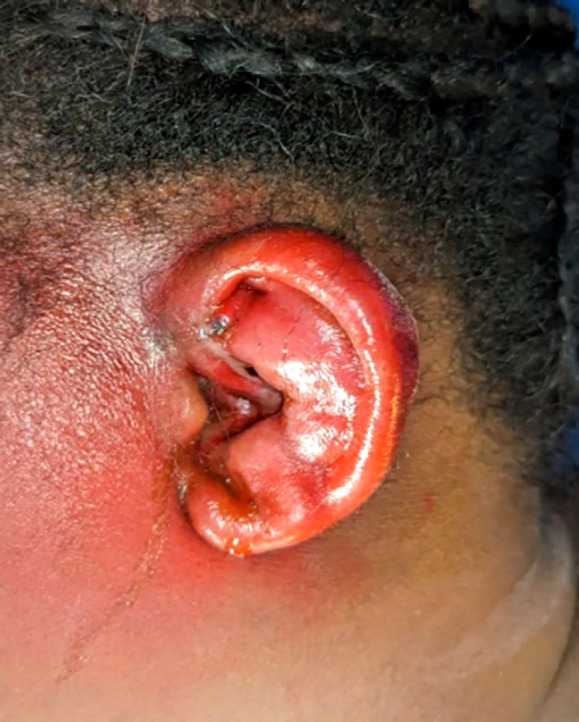
Périchondrite de l’oreille gauche Perichondrits of the left ear

Les pathologies infectieuses ont été retrouvées dans 19 cas, soit 11,9 % des pathologies auriculaires et périauriculaires, avec 17 cas de localisation auriculaire. Il s’agissait de périchondrites purulentes (Fig. 5) dans 18 cas et de zona dans un cas. Les périchondrites ont été traitées par drainage et antibiothérapie dans tous les cas. Une antibiothérapie à base de la ciprofloxacine par voie générale a été administrée pendant 10 jours.

**Figure 6:**
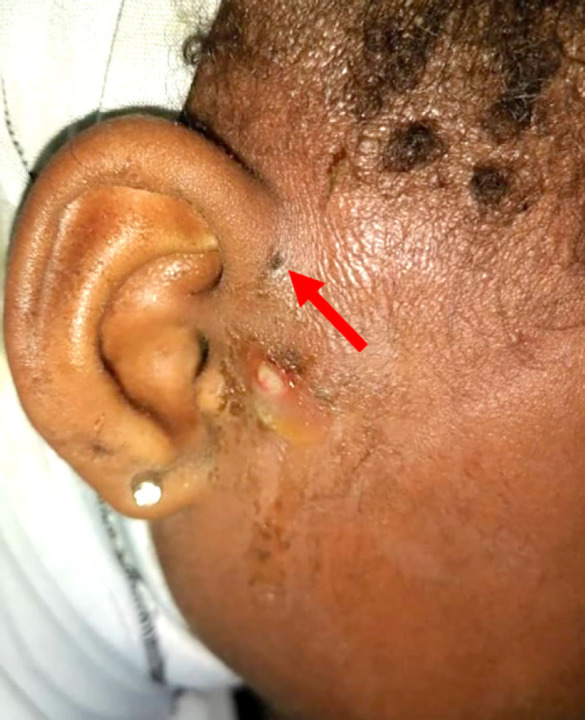
Fistule pré-hélicienne surinfectée de l’oreille droite (flèche rouge) Superinfected prehelical fistula of the right ear (red arrow)

Les pathologies congénitales ont été retrouvées dans 38 cas. Il s’agissait de fistules préhéliciennes dans 34 cas (Fig. 6), de pathologies périauriculaires, de microties (sans atteinte de l’oreille moyenne) dans 2 cas, d’une malformation du lobule dans un cas et d’une oreille décollée dans un cas. Le traitement a été chirurgical, à type d’exérèse pour les fistules et non précisé pour les microties et la malformation du lobule.

**Figure 7:**
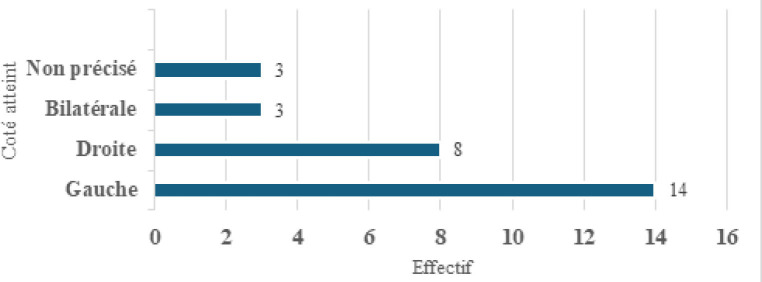
Répartition des chéloïdes selon le côté atteint Distribution of keloids by affected side

**Figure 8:**
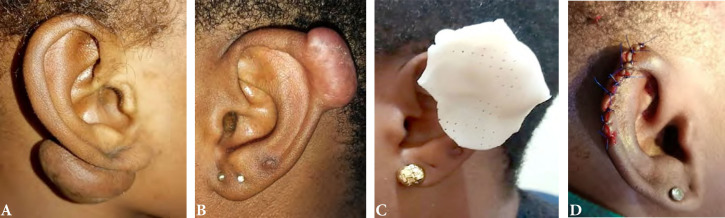
Chéloïdes de l’oreille Ear keloids

Les tumeurs et pseudotumeurs étaient représentées par les chéloïdes dans 28 cas (53 %), les kystes retro-auriculaires dans 22 cas (42 %), un cas de lipome, un cas de carcinome épidermoïde et un cas de chondrome. Pour les chéloïdes, un antécédent de piercing a été retrouvé dans 6 cas, un traumatisme dans 3 cas et non précisé dans 19 cas. Le siège de la chéloïde était le lobule et l’hélix dans 10 cas chacun et non précisé dans huit cas, le côté gauche était retrouvé dans 14 cas soit 50 % (Fig. 7). Le traitement a consisté en une exérèse des lésions, suivie d’infiltrations de corticoïdes dans tous les cas des chéloïdes. Un cas a bénéficié d’une pressothérapie qui s’est soldée par un échec conduisant à l’exérèse chirurgicale (Fig. 8).

## Discussion

Dans la littérature, les études concernant les pathologies auriculaires et/ou périauriculaire sont rarement rapportées. Les lésions de l’oreille externe sont les plus décrites, avec une prédominance des pathologies du conduit auditif [1, 3, 8]. Les pathologies auriculaires sont dominées par les traumatismes du lobule et de l’hélix, comme rapporté dans la littérature [14]. La densité croissante du parc automobile ainsi que l’augmentation des violences justifient les circonstances de survenue qui sont les coups et blessures volontaires et les accidents de la voie publique [5, 13]. La position et l’exposition anatomique de l’auricule justifie cette atteinte traumatique et la reconstruction avec respect des reliefs anatomiques reste le défi du chirurgien [16]. Les othématomes post-traumatiques sont un autre type de lésion auriculaire et ils ont été retrouvés dans six cas. Non traités, ils peuvent évoluer vers la surinfection de l’hématome, voire la nécrose cartilagineuse. Le drainage avec mise en place d’un bourdonnet de compression a été l’attitude chirurgicale adoptée dans notre pratique, comme recommandé par certains auteurs [6, 12].

Les chéloïdes ont représenté 53 % des tumeurs et pseudotumeurs dans notre étude. Le piercing est la cause la plus souvent rapportée, avec une prédominance féminine à cause d’un souci esthétique [7]. La prise en charge des chéloïdes varie selon les auteurs.

Nous avons procédé à l’exérèse avec infiltration de corticoïdes. Cette technique donne de bons résultats selon certains auteurs qui recommandent une association thérapeutique de la chirurgie, l’infiltration et la pressothérapie [7, 11]. Nous avons procédé dans un cas à la pressothérapie avec échec thérapeutique et finalement à la prise en charge chirurgicale. Les fistules préhéliciennes ont représenté 89 % des pathologies périauriculaires. Elles sont souvent diagnostiquées en ORL ou en dermatologie, dans leurs formes symptomatiques ou non [4]. Mais la prise en charge chirurgicale est indiquée devant les formes symptomatiques, comme les surinfections et les abcès [15]. Dans notre contexte, les patients ont consulté, car confrontés à des épisodes de surinfections. L’exérèse chirurgicale a été faite à distance de l’épisode infectieux.

## Conclusion

Cette étude épidémiologique et descriptive des pathologies auriculaires et périauriculaires au service d’ORL du CHU SO de Lomé a montré que les pathologies du pavillon étaient les plus fréquentes, avec une prédominance des lésions pseudotumorales, traumatiques et congénitales. La prise en charge était fonction du type de lésion. L’étude plus détaillée des séquelles fonctionnelles et esthétiques constitue notre perspective.

## Contributions des auteurs

PEKOULA G, AMANA E ont procédé à la collecte des données et à la rédaction du document, REOULEMBAYE D H, ZEYI KR, FOMA W, AMANA B ont relu le document.

## Conflit d’intérêt

Les auteurs déclarent ne pas avoir de conflit d’intérêt.
